# A multi-modal longevity protocol integrating lifestyle, supplements, and autologous pro-regenerative cell-conditioned media: a pilot study

**DOI:** 10.3389/fragi.2026.1732426

**Published:** 2026-06-25

**Authors:** Esteban Ortega, Guenole Addor, Sonam Bhatia, Max Lewinsohn, Pierre-Edouard Sottas

**Affiliations:** 1 Wellbeing International Foundation, Hamilton, Bermuda; 2 xLongevity, Baar, Switzerland

**Keywords:** age reversal, autologous conditioned media, biological age, epigenetic clock, iron metabolism

## Abstract

**Introduction:**

Biological aging is a modifiable process contributing to functional decline and chronic disease risk. Multi-modal interventions targeting aging hallmarks have shown promise, yet human data integrating lifestyle, supplementation, and regenerative therapies remain limited.

**Methods:**

This single-arm, open-label pilot trial in 16 healthy adults aged 46-72 (14 completers) tested a 17-week multi-modal program integrating lifestyle optimization, targeted supplementation, and two intravenous infusions of autologous pro-regenerative cell-derived conditioned media (APRC-CM). Trial registration: ClinicalTrials.gov, NCT07322224.

**Results:**

The intervention improved clinical biomarkers and reduced biological-age measures (PhenoAge and an epigenetic DNA-methylation clock) despite the cohort’s already healthy baseline (chronological age 59.3 years; PhenoAge 55.5 years; epigenetic age 57.9 years), consistent with a net reversal of biological age rather than merely a correction of age acceleration. PhenoAge declined by 2.0 years (p = 0.014), reaching 5.8 years below chronological age; epigenetic age decreased by 2.7 years (p = 0.003), widening the gap to 4.1 years. Exploratory response analysis revealed heterogeneity, with a larger-response subgroup showing a mean epigenetic-age reduction of 5.1 years versus 0.2 years in a smaller/no-response subgroup. Among baseline biomarkers, serum iron strongly predicted response: lower iron correlated with greater epigenetic-age reduction (r = 0.64; p = 0.013), identifying baseline iron status as a candidate prognostic biomarker of response. No adverse events were reported.

**Discussion:**

Such reductions in health-optimized individuals are notable and may indicate a lowering below expected norms rather than solely a correction of age acceleration. The observed response heterogeneity, together with baseline iron metabolism as a potential predictor of response, is hypothesis-generating and warrants validation in larger, controlled trials, particularly as this open-label, single-arm pilot lacked a control or sham comparator.

## Introduction

Biological aging is characterized by a progressive decline in cellular and physiological functions, contributing to increased susceptibility to chronic diseases, loss of resilience, and elevated mortality risk (López-Otín et al., 2013; Kennedy et al., 2014). Unlike chronological aging, biological aging is influenced by modifiable cellular and molecular mechanisms, thereby offering potential targets for therapeutic interventions aimed at extending healthspan and reducing disease incidence (Horvath, 2013; Levine et al., 2018).

Recent advancements in longevity research indicate that isolated therapeutic approaches, although promising, may fail to comprehensively address the multi-dimensional complexity of aging (Cesari et al., 2013). Contemporary models increasingly support multimodal, preventive, and regenerative approaches targeting metabolic and cellular drivers of aging (Boccardi, 2024), reinforcing the rationale for integrated, multi-domain interventions.

Remarkable successes in biological age reversal have already been achieved in animal models, with recent studies demonstrating near-complete rejuvenation of biological age in rats using plasma-derived exosome therapies (Horvath et al., 2024). In humans, translation remains at an early stage; however, initial studies have reported encouraging results. The TRIIM trial demonstrated a 2.5-year reduction in biological age following a 12-month intervention combining growth hormone, metformin, DHEA, zinc, and vitamin D3 (Fahy et al., 2019). An 8-week lifestyle-based program incorporating diet, exercise, sleep optimization, and supplementation resulted in a 3.23-year reduction compared to controls (Fitzgerald et al., 2021; Fitzgerald et al., 2022; Fitzgerald et al., 2024), and a fasting-mimicking diet protocol applied over 3 months achieved a 2.5-year reduction (Brandhorst et al., 2024). However, to date, human studies have rarely combined structured lifestyle optimisation and targeted supplementation with an autologous, intravenously delivered conditioned-media regenerative component within a single protocol while tracking biological age with validated clocks. We therefore designed the intervention to combine upstream behavioural optimisation (lifestyle), supportive metabolic inputs (supplementation), and an autologous regenerative signalling component (APRC-CM) to act in a complementary manner on biological-age readouts.

Among emerging regenerative approaches, conditioned media derived from autologous pro-regenerative cells (APRC-CM)—including hematopoietic progenitors, mesenchymal progenitors, endothelial progenitors, and supportive immune cells—have demonstrated potent regenerative, anti-inflammatory, and senolytic properties (Sriramulu et al., 2018; Gunawardena et al., 2019). APRC-CM contains a rich mixture of extracellular vesicles, cytokines, growth factors, anti-inflammatory mediators, and microRNAs, collectively capable of modulating intercellular signaling, enhancing tissue repair, reducing chronic inflammation, and mitigating cellular senescence (Mintz et al., 2014; Huang et al., 2021). Specifically, using autologous pro-regenerative cell-derived conditioned media confers additional clinical advantages by reducing risks of immune rejection, improving biocompatibility, and circumventing the regulatory and safety challenges typically associated with allogeneic or cell-based therapies (Baldari et al., 2017; Mitchell et al., 2019; Giannasi et al., 2020).

Building on these advances, we conducted a pilot study of a multi-modal intervention combining lifestyle optimisation and targeted supplementation with APRC-CM as an autologous regenerative signalling component. In our study, APRC-CM refers specifically to the conditioned media obtained from a selected subpopulation of autologous peripheral blood mononuclear cells (PBMCs), including hematopoietic and immune progenitors, but not mesenchymal stromal cells, which were not isolated or cultured in our protocol. The primary objective was to assess the safety and tolerability of the protocol through standard clinical biomarkers. Secondary objectives included evaluating the intervention’s preliminary efficacy in modulating biological aging, as measured by validated epigenetic and phenotypic clocks.

## Methods

This was a single-arm, open-label pilot study conducted over 17 weeks to explore the impact of a multi-modal intervention combining lifestyle optimisation, targeted supplementation, and APRC-CM therapy on clinical and biological ageing markers. Participants were healthy volunteers aged 45–75 years recruited according to predefined eligibility criteria. Participants were recruited through direct contact within the investigators’ longevity and health-optimization networks. Inclusion criteria consisted of generally good health confirmed by comprehensive medical history screening and baseline clinical evaluations, and willingness and ability to adhere to the study interventions for the full study duration. Exclusion criteria included significant chronic illnesses (such as advanced cardiovascular disease, active malignancy, uncontrolled diabetes, or severe respiratory disorders), hospitalization or emergency medical interventions within the prior year, participation in anti-ageing interventions or treatments in the preceding 6 months, and known hypersensitivity or allergies to any intervention components. Participants provided written informed consent after receiving comprehensive information regarding the study objectives, procedures, potential risks, and benefits, including the investigational nature of intravenous APRC-CM and foreseeable infusion-related risks. Administration was performed under medical supervision in a clinical setting with monitoring and adverse-event documentation according to the protocol. Trial registration: ClinicalTrials.gov, NCT07322224.

Lifestyle optimization encompassed dietary counseling, physical activity guidance, and sleep hygiene recommendations. Participants received individualized 1-h coaching sessions at Weeks 1, 3, and 9, delivered either in person or through a telemedicine platform. Between scheduled sessions, participants had access to their coaches for additional support via text messaging. Dietary counseling emphasized a whole-food, anti-inflammatory diet with a daily protein intake of >1.5 g/kg body weight and simple carbohydrates restricted. A daily intermittent fasting regimen, aiming for at least 16 h of fasting for male participants and at least 12 h for female participants throughout the study duration was recommended. The shorter fasting target for female participants was selected as a conservative sex-informed tolerability choice given sex-specific differences in intermittent-fasting responses. Physical activity goals included achieving ∼10,000 steps per day and engaging in structured resistance training at least twice weekly. Sleep recommendations included maintaining a consistent sleep-wake schedule (∼seven to eight h/night), exposure to morning sunlight (∼10 min/day), and limiting evening blue-light exposure.

Participants received the same predefined supplementation protocol throughout the 17-week intervention. Nicotinamide mononucleotide (NMN, 500 mg per scoop) was administered sublingually once daily. All other supplements were taken orally. The daily regimen included collagen peptides (10 g/day), GlyNAC powder providing glycine (5 g/day) and N-acetylcysteine (1 g/day), creatine monohydrate (5 g/day), taurine (1 g/day), liposomal curcumin (600 mg/day), liposomal coenzyme Q10 (150 mg/day), and nucleotides (750 mg/day). All supplements were manufactured according to GMP standards and were provided to participants at baseline to standardise the intervention and support adherence throughout the study period. Adherence was not formally quantified; compliance was encouraged and assessed qualitatively through regular check-ins and ongoing participant contact. All supplements were sourced from xLongevity Center AG, Switzerland, with the exception of the nucleotides, which were supplied by PKN AG, Switzerland.

At baseline (Week 1), 100–150 mL of peripheral blood was collected per participant for preparation of APRC-CM. The protocol involved isolation of an autologous PBMC-derived cell fraction without subsequent cell expansion, followed by controlled *ex vivo* conditioning under predefined environmental conditions to generate conditioned media enriched with extracellular vesicles, cytokines, growth factors, anti-inflammatory mediators, and microRNAs. All APRC-CM preparations were produced per participant by an independent, internationally accredited facility using a standardised, closed and batch-controlled workflow comprising (i) isolation of a defined autologous PBMC-derived input from whole blood, (ii) controlled *ex vivo* conditioning, with conditioning duration, temperature, volume, PBMC input range, and EV output recorded for each participant as part of the batch documentation and release process, and (iii) harvest, clarification, and sterile final fill of the resulting conditioned medium. Lot documentation ensured full chain-of-identity/chain-of-custody and traceability of processing windows, in-process holds, and storage/transport conditions. Release followed routine quality control testing appropriate for autologous conditioned-media products, including validated aseptic processing controls, predefined cell input acceptance ranges, quantified extracellular vesicle (EV) output, predefined acceptance criteria, deviation handling, and retained aliquots for confirmatory testing. Participants received intravenous infusions of APRC-CM under clinical supervision at Weeks 3 and 10. Final assessments were conducted at Week 17, 7 weeks after the last infusion, providing a post-intervention follow-up window for safety monitoring and outcome evaluation.

The primary outcome was change in epigenetic biological age from baseline to week 17, with change in phenotypic age (PhenoAge) as a key secondary outcome. Safety was monitored throughout via adverse-event reporting and routine clinical laboratory assessments (haematology and chemistry panels, liver and kidney function). Additional laboratory biomarkers (efficacy/safety) were analysed as exploratory outcomes to characterise physiological changes and generate hypotheses regarding determinants of response. Given the pilot nature, small sample size, and exploratory intent of the broader biomarker panel, we did not apply correction for multiple comparisons; nominal p-values are reported and should be interpreted as hypothesis-generating rather than confirmatory.

Blood samples were collected by venipuncture at baseline (Week 1) and at the end of the intervention (Week 17) under standardized fasting conditions. Clinical laboratory analyses included complete blood count, liver function tests, kidney function tests, fasting glucose, inflammatory biomarkers, and lipid profiles.

PhenoAge was calculated from nine clinical biomarkers and chronological age following the original algorithm (Levine et al., 2018), providing a composite measure of phenotypic aging validated against morbidity and mortality. In addition, dried blood spot samples were collected for DNA methylation analysis, with biological age estimated using OMICmAge, a multi-omic epigenetic clock trained on CpG methylation sites (Chen et al., 2023). Biological age analyses were conducted by two independent third-party laboratories (PhenoAge and DNA methylation clocks) using coded samples. The laboratories had no access to participant identity or sampling timepoint (baseline vs. post-intervention); unblinding occurred only after completion of data processing, QC, and clock calculations.

## Results

A total of 16 participants were enrolled in the study. Two withdrew before completion due to work-related obligations, resulting in a final cohort of 14 participants (7 females, seven males) who completed the 17-week protocol and were included in the analysis. The mean chronological age at baseline was 59.3 years (range: 46.9–72.4).

Baseline and post-intervention results for hematological parameters (including red cell indices: Hgb, Hct, RBC, MCV, MCH, MCHC, RDW), platelets (PLT, MPV), white blood cell differentials (WBC, Neu%, Lym%, Mon%, Eos%, Bas%), inflammatory markers (hs-CRP, ESR), electrolytes (Na, K, Cl, HCO_3_, Ca, phosphate), renal function markers (creatinine, urea, uric acid), liver enzymes (ALP, AST, ALT, GGT, bilirubin), tissue damage marker (LDH), protein metabolism (total protein, albumin, globulin), glucose metabolism (fasting glucose), lipid profile (cholesterol, triglycerides, LDL, HDL, non-HDL), iron metabolism (serum iron, TIBC, transferrin, ferritin), thyroid function (TSH, free T4), and vitamin D are summarized in Table 1.

**TABLE 1:** Comparison of baseline (pre-intervention) and final (post-intervention) biochemical and hematological parameters.

Blood biomarkers	Baseline (mean ± SD)	Final (mean ± SD)	p-value
ALP (IU/L)	71.64 ± 14.96	80.36 ± 15.24	0.0070
ALT (IU/L)	25.86 ± 10.11	22.29 ± 7.00	0.18
AST (IU/L)	26.86 ± 5.36	26.36 ± 7.90	0.76
Albumin (g/L)	44.00 ± 1.71	45.00 ± 2.63	0.26
Bas (x10^9/L)	0.04 ± 0.01	0.04 ± 0.01	0.87
Bilirubin (umol/L)	9.14 ± 2.77	8.07 ± 3.69	0.18
CK (IU/L)	106.21 ± 49.11	173.57 ± 203.69	0.19
hsCRP (mg/L)	2.75 ± 2.69	2.27 ± 1.97	0.39
Calcium (mmol/L)	2.32 ± 0.06	2.37 ± 0.07	0.039
Chloride (mmol/L)	103.86 ± 1.99	103.21 ± 1.81	0.30
Cholesterol (mmol/L)	4.93 ± 0.88	5.15 ± 0.86	0.073
Creatinine (umol/L)	77.50 ± 16.32	80.86 ± 22.29	0.20
ESR (mm/hr)	7.07 ± 5.15	6.86 ± 5.16	0.89
Eos (x10^9/L)	0.18 ± 0.13	0.16 ± 0.12	0.66
Ferritin (ug/L)	143.00 ± 117.66	135.36 ± 117.27	0.49
Free Thyroxine (pmol/L)	16.09 ± 1.96	15.67 ± 1.60	0.25
GGT (IU/L)	24.86 ± 11.65	21.71 ± 9.00	0.047
Globulin (g/L)	24.93 ± 1.98	25.07 ± 2.67	0.73
HCO3 (mmol/L)	23.71 ± 2.73	24.36 ± 2.59	0.50
HCT (L/L)	0.41 ± 0.02	0.42 ± 0.03	0.59
HDL (mmol/L)	1.49 ± 0.29	1.49 ± 0.29	0.99
Hb (g/L)	142.29 ± 8.91	142.14 ± 12.82	0.96
Iron (umol/L)	17.97 ± 3.67	18.44 ± 6.34	0.79
LDH (IU/L)	213.71 ± 27.81	196.36 ± 24.57	0.025
LDL (mmol/L)	2.91 ± 0.79	3.05 ± 0.80	0.23
Lym (x10^9/L)	1.93 ± 0.42	1.96 ± 0.62	0.78
MCH (pg)	30.81 ± 2.81	30.21 ± 2.75	<0.001
MCHC (g/L)	343.86 ± 9.05	339.57 ± 9.47	0.057
MCV (fL)	89.52 ± 6.77	88.86 ± 7.17	0.33
MPV (fL)	10.59 ± 0.75	10.62 ± 0.91	0.72
Mon (x10^9/L)	0.48 ± 0.11	0.51 ± 0.11	0.36
Neu (x10^9/L)	3.39 ± 0.78	3.63 ± 0.91	0.20
Non-HDL (mmol/L)	3.44 ± 0.96	3.66 ± 0.93	0.073
Phosphate (mmol/L)	1.00 ± 0.13	1.09 ± 0.18	0.089
Platelet (x10^9/L)	245.50 ± 38.13	260.71 ± 47.92	0.027
Potassium (mmol/L)	4.12 ± 0.32	4.33 ± 0.24	0.034
RBC (x10^12/L)	4.65 ± 0.52	4.73 ± 0.39	0.52
RDW (%)	13.59 ± 1.68	13.63 ± 1.61	0.77
Fasting glucose (mmol/L)	5.51 ± 0.47	5.16 ± 0.92	0.13
Sodium (mmol/L)	141.14 ± 2.68	140.86 ± 1.23	0.62
TIBC (umol/L)	56.86 ± 8.86	56.86 ± 7.36	0.99
TSH (mIU/L)	1.87 ± 0.68	1.72 ± 0.59	0.23
Total protein (g/L)	69.07 ± 2.06	70.07 ± 3.95	0.31
Transferrin Sat (%)	32.07 ± 6.67	32.71 ± 11.44	0.85
Triglycerides (mmol/L)	1.14 ± 0.54	1.33 ± 0.63	0.16
Urea (mmol/L)	5.22 ± 1.71	5.29 ± 1.66	0.82
Uric acid (umol/L)	292.21 ± 48.15	293.29 ± 43.25	0.91
Vit D (nmol/L)	65.21 ± 26.06	60.00 ± 29.10	0.21
WBC (x10^9/L)	6.01 ± 0.85	6.26 ± 1.37	0.32

Values are presented as mean ± standard deviation. Wilcoxon Signed-Rank tests were conducted to evaluate the statistical significance of changes between time points.

Due to the small sample size (n = 14) and non-normal distributions in several biomarkers, the Wilcoxon Signed-Rank test was used to assess pre-post changes. A two-tailed, nominal (unadjusted) p-value <0.05 was considered statistically significant; given the pilot nature and exploratory scope of the biomarker panel, no correction for multiple comparisons was applied and results should be interpreted as hypothesis-generating rather than confirmatory.

Among hematological parameters, MCH significantly decreased after the intervention (p < 0.001), although all values remained within reference ranges except for one participant with minor beta-thalassemia. Platelet count increased significantly (p = 0.027), with all values remaining within physiological limits. In the electrolyte panel, potassium (p = 0.034) and calcium (p = 0.039) both increased significantly, also remaining within reference ranges. In the liver panel, ALP increased (p = 0.007) and GGT decreased (p = 0.047), with no values outside physiological limits. LDH decreased significantly (p = 0.025), remaining within range for all participants.

Creatinine showed no significant change overall (p = 0.12), but one participant with elevated baseline creatinine (114 μmol/L) experienced a further increase to 135 μmol/L post-intervention, also prompting follow-up. hs-CRP showed a non-significant overall decrease (p = 0.39); however, the distribution shifted markedly, with four participants above the 5 mg/L threshold at baseline and only one remaining above this level after the intervention, suggesting heterogeneous but directionally consistent improvements in low-grade inflammation. All other biomarker values remained within physiological reference ranges, suggesting no new clinically relevant abnormalities at the group level in this already very healthy cohort.

Biological age results are presented in Table 2. At baseline, the mean PhenoAge was 55.5 years, 3.8 years younger than chronological age (nominal p = 0.038), reflecting a selection bias toward particularly healthy individuals. After the intervention, PhenoAge decreased to 53.9 years, extending the gap to 5.8 years. This corresponds to a net reduction of 2.0 years in biological age (p = 0.014).

**TABLE 2:** Individual baseline and final chronological and biological age estimates for the 14 study participants, one row per participant.

ID	Baseline			Final			Final BA gap		ΔBA gap	
	Age	PA	OA	Age	PA	OA	PA	OA	ΔPA	ΔOA
1	47.0	59.9	42.1	47.4	57.8	38.3	10.4	−9.1	−2.5	−4.2
2	58.0	49.8	63.5	58.4	51.9	58.7	−6.5	0.3	1.7	−5.2
3	57.8	50.1	61.4	58.2	53.0	57.3	−5.2	−0.9	2.5	−4.5
4	66.1	70.5	70.4	66.5	69.9	65.6	3.4	−0.9	−1.0	−5.2
5	64.4	53.6	64.5	64.8	51.8	64.4	−13.0	−0.4	−2.2	−0.5
6	56.9	51.1	55.9	57.4	46.3	50.9	−11.1	−6.5	−5.3	−5.5
7	62.0	53.8	51.1	62.4	51.4	51.5	−11.0	−10.9	−2.8	0.0
8	64.5	60.8	56.8	64.9	59.2	55.2	−5.7	−9.7	−2.0	−2.0
9	51.7	51.5	50.0	52.0	52.3	52.2	0.3	0.2	0.5	1.9
10	49.4	38.9	43.7	49.8	36.0	43.3	−13.8	−6.5	−3.3	−0.8
11	58.1	53.6	58.6	58.4	51.9	52.7	−6.5	−5.7	−2.0	−6.2
12	72.5	67.8	69.9	72.9	63.3	69.6	−9.6	−3.3	−4.9	−0.7
13	70.6	68.6	71.5	70.9	63.6	66.6	−7.3	−4.3	−5.3	−5.2
14	51.6	46.9	51.1	51.9	46.7	51.8	−5.2	−0.1	−0.5	0.4
Mean	59.3	55.5	57.9	59.7	53.9	55.6	−5.8	−4.1	−2.0	−2.7

BA, biological age; PA, PhenoAge; OA, OMICmAge; Δ, change from baseline to final. Final BA, gap is calculated as final BA minus final chronological age; negative values indicate BA, below chronological age; ΔBA, gap is calculated as final BA gap minus baseline BA, gap. The final row reports cohort means.

One participant with minor beta-thalassemia had chronically elevated RDW (19.0% baseline, 18.6% post). Because RDW is a component of the PhenoAge algorithm, and RDW is chronically altered in beta-thalassemia for reasons unrelated to biological aging, PhenoAge is not an appropriate biological-age estimate for this participant. This individual was the only participant whose PhenoAge exceeded chronological age at the end of the study. As a sensitivity analysis, exclusion of this participant increased the post-intervention PhenoAge gap to 7.0 years among the remaining 13 participants.

For the epigenetic clock, the mean biological age at baseline was 57.9 years, 1.4 years below chronological age (nominal p = 0.29). After the 17-week intervention, it decreased to 55.6 years, representing a 4.1-year gap and a net reduction of 2.7 years in epigenetic age (p = 0.003).

To assess response heterogeneity, we first examined the distribution of individual changes in biological age. PhenoAge changes were relatively uniform, with a bimodality coefficient of 0.44 and no evidence of bimodality in an exploratory likelihood ratio test comparing one- and two-component Gaussian mixture models (p = 0.84). In contrast, epigenetic-age changes showed stronger separation, with a bimodality coefficient of 0.74 and support for a two-component structure in the exploratory likelihood ratio test (p = 0.0049). Based on this exploratory model, participants separated into a larger-response subgroup (n = 7; mean epigenetic-age reduction of 5.1 years) and a smaller/no-response subgroup (n = 7; mean reduction of 0.2 years). Given the small sample size, this subgroup structure should be interpreted as hypothesis-generating rather than confirmatory.

We then examined whether this response heterogeneity was associated with baseline biomarkers. Baseline serum iron was significantly associated with change in epigenetic age (r = 0.64, p = 0.013), indicating that lower baseline iron corresponded to greater epigenetic-age reduction, whereas higher baseline iron was associated with little or no response. Ferritin showed a similar trend (r = 0.53, p = 0.054), and transferrin was moderately associated (r = 0.48, p = 0.081). No other baseline biomarkers showed significant associations with epigenetic-age change; given the small sample size and restricted baseline variance in this health-optimised cohort, these null findings should be interpreted cautiously.

## Discussion

This 17-week intervention in a cohort of healthy middle-aged adults resulted in measurable improvements across several biological domains, including statistically significant changes in multiple blood-based biomarkers and reductions in both PhenoAge and epigenetic biological age. Importantly, these effects were achieved in an already health-optimized group, as evidenced by values within clinical reference ranges across nearly all markers at baseline.

Among conventional biomarkers, we observed significant post-intervention changes in MCH, platelets, potassium, calcium, ALP, GGT and LDH. These changes, while remaining within physiological ranges, suggest subtle shifts in metabolic and cellular activity. For example, the increase in platelet count and decrease in LDH may reflect reduced low-grade inflammation or improved cellular turnover. The decrease in fasting glucose, though modest, aligns with improved glycemic regulation.

One of the most striking findings was the consistent reduction observed across both biological-age metrics, reaching magnitudes exceeding those reported in prior human studies of comparable duration. PhenoAge decreased by an average of 2.0 years and epigenetic age by 2.7 years over the 17-week period, despite participants already presenting with biologically younger profiles at baseline (e.g., PhenoAge 3.8 years below chronological age). When the participant with minor beta-thalassemia—known to interfere with RDW and thus with the PhenoAge algorithm—was excluded, the post-intervention PhenoAge gap increased to 7.0 years, underscoring the sensitivity of this metric to hematologic outliers. Importantly, these reductions occurred in a cohort that was already healthier than average, in contrast to most previous studies where decreases in biological age were observed primarily in populations with metabolic disorders, micronutrient deficiencies, or other conditions associated with accelerated aging. In those contexts, reported improvements may reflect a correction toward expected biological age. By contrast, in the present study, biological age was reduced further below chronological age, with the cohort on average reaching a gap of 7 years younger biologically than their chronological age, and several individuals showing reductions exceeding 10 years. This suggests that multi-modal interventions may achieve measurable decreases in biological age even in already healthy individuals, extending beyond simple normalization of accelerated aging. It should also be noted that short-term changes in epigenetic clocks remain open to interpretation. Although these clocks are associated with long-term health outcomes at the population level, it is not yet clear whether short-term shifts reflect durable biological rejuvenation or transient methylation changes. Longer-term follow-up will be needed to determine whether the reductions observed here are sustained.

Interestingly, while the average reductions in both biological age measures were statistically significant, further analysis revealed distinct patterns of response. For PhenoAge, the distribution of changes was consistent with a unimodal pattern, suggesting a relatively homogeneous effect across participants. In contrast, epigenetic-age changes showed greater response heterogeneity, with an exploratory two-component structure consistent with larger-response and smaller/no-response subgroups. The magnitude of this divergence further underscores the importance of individual variability: while the overall cohort exhibited a meaningful reduction in epigenetic age, the larger-response subgroup experienced an average decrease of 5.1 years, compared to 0.2 years in the smaller/no-response subgroup. These findings highlight the need for biomarker-driven stratification and support the development of personalized approaches to optimize the effectiveness of longevity interventions.

As the intervention was multi-modal, the observed effects may reflect contributions from lifestyle optimisation, supplementation, APRC-CM, or their combination. Lifestyle and supplementation may improve metabolic/inflammatory tone and mitochondrial/redox capacity, while APRC-CM provides an autologous signalling milieu; however, this pilot design does not permit attribution of effects to individual components.

The significant association between baseline iron levels and treatment response is consistent with the hypothesis that iron metabolism may modulate the biological effects of this multi-modal intervention. High iron levels are known to promote oxidative stress, inflammation, and ferroptosis, which could plausibly interfere with the intended biological effects of the programme. In particular, excess iron could counteract anti-inflammatory and mitochondrial-supportive effects of supplements such as NMN, GlyNAC, CoQ10, and curcumin, and may also attenuate cellular responsiveness to APRC-CM signalling. Because the conditioned media originate from a heterogeneous population of regenerative cells that includes haematopoietic progenitors, whose proliferation and differentiation are tightly regulated by intracellular iron availability, elevated systemic iron could plausibly influence the composition or activity of the resulting secretome. Iron overload has been reported to induce premature senescence and ferroptotic vulnerability in haematopoietic progenitors, which could reduce the pro-regenerative signalling conveyed through APRC-CM. Furthermore, several epigenetic-modifying enzymes are iron-dependent, and dysregulation under iron overload could, in principle, affect epigenetic remodelling capacity. Notably, iron-related biomarkers (serum iron, ferritin, transferrin) were not significantly correlated with epigenetic age at baseline, suggesting that iron status may relate more to response heterogeneity than to baseline biological age. Overall, these findings support baseline iron status as a candidate prognostic biomarker for response to the intervention, although mechanistic interpretations remain speculative and require confirmation in larger, controlled studies. Beyond baseline iron status, the study was not powered to robustly characterise determinants of responder/non-responder status, and additional predictors should be evaluated in larger cohorts.

Although hs-CRP did not show a statistically significant mean reduction, the shift from four participants above 5 mg/L at baseline to only one post-intervention suggests meaningful biological improvement in low-grade inflammation. This pattern, while not captured by average-based statistics, supports the value of examining distributional changes and individual trajectories.

This proof-of-concept study has several important limitations that must be considered when interpreting the findings. First, the absence of a control group limits the ability to definitively attribute observed changes to the intervention itself. Without a randomized comparison, improvements may reflect placebo effects, seasonal variation, regression to the mean, or other non-specific influences unrelated to the protocol. In addition, the open-label design introduces expectancy bias, and behavioural changes driven by participation/monitoring cannot be disentangled from intervention-specific effects. Future studies should incorporate randomised control conditions, including sham infusion comparators where feasible, to mitigate these sources of bias.

Second, the sample size was small (n = 14), limiting statistical power and the ability to detect subtle or subgroup-specific effects. While some changes reached statistical significance, others may have been underestimated due to insufficient power. The small cohort size also restricts generalizability to broader populations and precludes detailed stratified analyses by age, sex, or baseline health status.

Third, the cohort consisted of highly health-conscious individuals who were already biologically younger than their chronological age at baseline. Participant characterization focused primarily on clinical eligibility and laboratory biomarkers; socioeconomic variables, formal disability status, menopausal status, intervention-start season, and objective adherence measures were not systematically recorded and therefore could not be evaluated as predictors of response. This selection bias likely attenuated the magnitude of observed changes and makes it difficult to extrapolate findings to more heterogeneous, less motivated, or less health-optimized populations. Effects may differ in cohorts with higher baseline inflammation, metabolic dysfunction, or comorbidity burden, and should be evaluated in larger, more diverse populations with stratification by baseline risk profiles.

Fourth, the duration of the intervention and follow-up (17 weeks) was relatively short. While reductions in biological age were observed over this period, substantially greater than the duration of the intervention itself, the long-term durability and clinical significance of these changes remain uncertain.

Fifth, the intervention was multi-modal by design, integrating lifestyle optimization, targeted supplementation, and autologous conditioned media therapy. While this reflects a real-world longevity strategy, it limits the ability to isolate the individual contribution of each component. Future studies using factorial or stepwise designs, including subsets of the intervention components, are needed to disentangle which elements drive specific biological effects.

Finally, a limitation of the study was the absence of validated quality-of-life (QoL) measures. Although not formally assessed, participants spontaneously reported subjective improvements such as increased energy, reduced fatigue, a sense of feeling younger, less joint pain and stiffness (including relief of mild arthritic symptoms), improved mood and mental wellbeing, and renewed motivation to engage in physical activity. These consistently reported improvements suggest effects on perceived wellbeing that may not be fully captured by biomarker-based endpoints. Future studies should incorporate validated QoL questionnaires and functional assessments (e.g., grip strength, cardiorespiratory fitness, cognitive performance) to more comprehensively assess participant-reported outcomes alongside biological and clinical measures.

In summary, this pilot study demonstrates that a multi-modal intervention combining lifestyle optimization, targeted supplementation, and autologous pro-regenerative cell-derived conditioned media is safe and can produce substantial reductions in biological age and improvements in systemic biomarkers, even among already healthy individuals. Such reductions in health-optimized cohorts are notable and highlight the potential of multi-modal interventions including APRC-CM to meaningfully impact biological age trajectories in humans. The observed heterogeneity in epigenetic age responses underscores the need for personalized approaches and further investigation into predictors of response. Baseline iron status emerged as a potential biomarker, suggesting that systemic iron status may be linked to response heterogeneity. Larger, randomized, double-blind clinical trials with multiple arms and incorporating standardized quality-of-life measures alongside biological age and clinical biomarkers will be essential to validate and expand these findings and clarify the specific contributions of each intervention component.

## Data Availability

The raw data supporting the conclusions of this article will be made available by the authors, without undue reservation.
